# Perspectives on blastomycosis in Canada in the face of climate change

**DOI:** 10.14745/ccdr.v50i11a04

**Published:** 2024-11-07

**Authors:** Amole Khadilkar, Lisa Waddell, Emily S Acheson, Nicholas H Ogden

**Affiliations:** 1 Environmental Public Health Division, Indigenous Services Canada, Ottawa, ON; 2 Public Health Risk Sciences, National Microbiology Laboratory Branch, Public Health Agency of Canada, Guelph, ON; 3 Public Health Risk Sciences, National Microbiology Laboratory Branch, Public Health Agency of Canada, Saint-Hyacinthe, QC; 4 Groupe de recherche en épidémiologie des zoonoses et santé publique (GREZOSP), Faculté de médecine vétérinaire, Université de Montréal, Montréal, QC

**Keywords:** blastomycosis, endemic, Canada, climate change, ecological niche, thermally dimorphic fungi, geographic distribution

## Abstract

Blastomycosis is a disease of potentially varied presentations caused by thermally dimorphic fungi that appear as mold at ambient temperatures and transform to yeast at body temperature. Inhalation of aerosolized fungal spores represents the primary mode of transmission. Exposure may follow outdoor activities that disturb soil, which is warm, moist, acidic and rich in organic debris, particularly within forested areas and in proximity to waterways. Blastomycosis is endemic to several parts of Canada, but is only reportable in Ontario and Manitoba, with Northwestern Ontario being considered a hyperendemic area with average annual incidence rates of over 25 cases per 100,000 population. Delays in diagnosis and treatment are frequently observed as the symptoms and imaging findings of blastomycosis may initially be mistaken for community-acquired pneumonia, tuberculosis or malignancy, which can result in interim disease progression and worsening clinical outcomes. Risks from fungal infections such as blastomycosis are likely to increase with climate change-associated shifts in temperature and rainfall, and this may contribute to the geographic expansion of cases, a phenomenon that appears to be already underway. Further research investigating the ecological niche of *Blastomyces* and its climate sensitivity could help facilitate better modelling of the potential impacts of climate change on risks to Canadians and inform more effective methods of exposure prevention. Early clinical recognition and treatment of blastomycosis remain the key to minimizing morbidity and mortality.

## Introduction

### Background

Blastomycosis is endemic to North America, particularly in areas bordering the Great Lakes, St. Lawrence Seaway and Mississippi and Ohio Rivers, though there is some evidence that the geographic distribution of blastomycosis is expanding beyond these historical margins ((1–4)). *Blastomyces dermatitidis* and *Blastomyces gilchristii* are the predominant fungal species that cause blastomycosis in North America ((5)). Sporadic cases attributed to *Blastomyces helicus* have also been reported in western Canada and the United States (US), though these cases are characterized by an atypical geographic range, mycological features and clinical epidemiology ((6–8)). In contrast to cases associated with *B. dermatitidis* and *B. gilchristii,* which most frequently involve immunocompetent individuals, *B. helicus* is opportunistic, mainly affecting those who are immunocompromised ((6–8)). Other species that have been described include 1) *B. percursus*, found in Africa and the Middle East; 2) *B. emzantsi*, found in South Africa; 3) *B. parvus*, reported to cause a rare pulmonary illness distinct from blastomycosis, called adiaspiromycosis, in North and South America, Eastern Europe and Australia; and 4) *B. silverae*, which has been identified in western Canada and is not currently known to cause disease in humans ((8–10)). *Blastomyces dermatitidis* and *B. gilchristii,* the more commonly encountered *Blastomyces* spp. in North America, are examples of thermally dimorphic fungi that grow as mold in the environment at ambient temperatures, but which, once spores and mold fragments are released into the air and inhaled, convert to thick-walled, broad-based budding yeast at body temperature in tissues, resulting in morbidity in approximately half of infected individuals ((11,12)).

### Objectives

As the burden of blastomycosis in Canada is likely under-recognized and under-reported, the objective of this overview was to synthesize the clinical and epidemiological evidence regarding blastomycosis in Canada to improve awareness about this disease, highlighting the potential impacts of climate change as well as current knowledge gaps and future directions.

## Methods

This literature review included a search for articles in PubMed and EBSCOhost databases using keywords related to blastomycosis and narrowed to literature conducted in North America from January 1, 2010, to June 9, 2024. The search was augmented by the evaluation of reference lists from the *Health of Canadians in a Changing Climate* report ((13)) and several relevant primary research studies and literature reviews (available upon request) to identify work omitted by the database search until we reached saturation. Grey literature was identified by implementing the search strategy in Google and screening the search results, stopping at the point where no new relevant results were identified on a page, and through targeted searches within the websites of key public health agencies in North America. Citations were screened for relevance and inclusion in the review with a focus on evidence from Canada. Where applicable, citations for older evidence were replaced by newer evidence to ensure this review was a good reflection of the most recent evidence on blastomycosis.

## Discussion

### Incidence and trends in Canada

Within Canada, blastomycosis has been detected in areas of Ontario, Québec, Manitoba, Saskatchewan, Alberta, Nova Scotia and New Brunswick, but is only a reportable disease in Ontario as of mid-2018 and in Manitoba since 2006 ((11,14–23)). In endemic areas, where the disease is reportable, incidence rates typically are 0.4–1.3 cases per 100,000 population per year ((22,24,25)); however, hyperendemic areas such as Northwestern Ontario, represented by the catchment area of the Northwestern Health Unit, have reported average incidence rates of over 25 cases per 100,000 population per year (range: 15–43 cases per 100,000 population per year) ((21,26–28)). In comparison, blastomycosis in the US is reportable in Arkansas, Louisiana, Colorado, Michigan, Minnesota and Wisconsin, where the annual incidence is two or fewer cases per 100,000 population, while hyperendemic areas in several northern counties of Wisconsin report annual rates in the range of 10–40 cases per 100,000 population ((29–31)). Given that blastomycosis is not a nationally notifiable disease either in Canada or the US, and in light of frequent asymptomatic infections and missed or delayed diagnoses, there is likely significant under-reporting of cases and under-estimation of its true incidence ((12,32–36)).

Although there has not been consistent surveillance in affected areas of Canada to establish definite trends in the incidence of blastomycosis, several studies suggest that cases have been increasing in some areas over time ((14,15,33,37)). The observed increases may be due to improving surveillance, increasing healthcare provider and/or community awareness of the infection, or possibly an expanding ecological niche for *Blastomyces* ((33,37,38)). It has been suggested that cases in dogs, whose incidence is estimated to be eight times greater than that of humans, may serve as sentinels for cases in humans ((39–42)). The high incidence in dogs could be related to the time spent outdoors, close contact with soil and such behaviours as digging and sniffing, which can increase exposure to fungal spores ((40)).

Epidemiological investigations suggest most human cases of blastomycosis are sporadic; however, a smaller proportion occur as outbreaks with epidemiologic links to a likely common source ((29,36,43,44)). Canadians are most commonly exposed during the warmer summer months when both the climate and human activities are more favourable for exposure; this leads to a majority of cases being diagnosed in the fall and winter, following an appropriate incubation period ((11,14,33,45–48)). In the US, no seasonal pattern was observed for blastomycosis-related hospital admissions during the period 2010–2020 ((49)).

### Ecological factors

The ecology of *Blastomyces* is not well understood due to challenges in conducting epidemiological investigations that aim to identify a potential source, given the long latency period of up to 15 weeks from exposure to clinical onset ((31)). Further challenges exist in recovering the fungus from the environment as *Blastomyces* is found to compete poorly with other microflora present in natural soil ((38,50–52)). Studies have reported the failure to repeat the isolation of *Blastomyces* at later time points from previously positive sample sites, suggesting that its growth can be sporadic and perhaps contingent on short-lived climactic and environmental conditions ((52,53)). Large outbreaks over an extended duration have been reported where optimal growth conditions are presumably stable, such as an outbreak spanning 10 weeks in Wisconsin in 2015 associated with recreational tubing along a river ((54)).

To date, research characterizing the environmental niche of *Blastomyces* suggests that moist soil with high organic content (e.g., rotting wood, decaying vegetation, animal manure) and acidic pH near lakes and rivers or in wooded areas is well suited for *Blastomyces* spp. survival ((52,53,55–58)). Laboratory and outbreak investigations also indicate that recent rainfall is important for the recovery of *Blastomyces* from environmental samples and the release of spores into the environment, which are then dispersed during windy weather ((52,56,59–62)). While outbreaks have occurred in the relative absence of rain too, other factors may have been present that created favourable conditions for transmission, such as proximity to waterways and/or soil-disrupting activity ((54,63)).

### Risk groups

Risk of exposure can increase with human activities that disturb soil and aerosolize spores, including occupations involving high-risk outdoor activities such as construction, excavation, landscaping and forestry work ((1,34,38,48,57,60,64–67)). Other exposures may come from outdoor recreational activities, including hunting, fishing, canoeing, camping, hiking and the use of all-terrain vehicles (ATVs), which involve close contact with soil and decaying vegetation near waterways ((1,38,46,55,64,68,69)).

The majority of clinical cases occur in adults (mostly middle-aged adults 30–59 years of age) with less than 13% occurring in children ((11,12,14,16,26,45,62,70)). Males are more frequently affected than females, which may reflect a greater likelihood of occupational or recreational environmental exposures ((38)); however, a sex-specific, possibly hormonally mediated, susceptibility to infection has been proposed as a factor in another endemic dimorphic mycosis that predominates in males, namely coccidioidomycosis ((71,72)).

Indigenous Peoples in Ontario and Manitoba have been found to be disproportionately affected by blastomycosis ((11,33,46)). Similarly, in the US, incidence rates among Native American and Alaska Native Peoples between 2010 and 2020 were approximately six times higher than in non-Hispanic Whites ((71)). A genetic predisposition may account for these findings, but alternate explanations include differences in occupational or recreational exposures, access to medical care, socioeconomic status and/or other social determinants of health ((12,36,38,71)). Higher rates of comorbidities and smoking among Indigenous Peoples in Northwestern Ontario were also offered as possible contributing factors ((33)).

### Clinical features of blastomycosis

The primary route of *Blastomyces* transmission is through the inhalation of aerosolized spores; however, infection has also been documented from direct cutaneous inoculation by traumatic injury (e.g., needlestick injuries in laboratory workers or veterinarians) or following a bite or scratch from an infected animal ((73–76)). Isolated case reports of possible sexual transmission appear in the literature, along with rare reports of perinatal transmission ((77–81)).

The incubation period of blastomycosis is estimated to be 30 to 45 days for inhalational exposure with a possible range of 14 to 106 days, whereas the incubation period for primary cutaneous inoculation is approximately two weeks ((26,54,55,73,78)).

The clinical spectrum of disease includes subclinical infection in approximately 50% of cases; acute pneumonia, indistinguishable from community-acquired bacterial pneumonia; chronic pneumonia, mimicking tuberculosis (cavitary lesions, miliary pattern) or malignancy (nodules, masses); and, at the most severe end of the spectrum, lung infection may progress to acute respiratory distress syndrome (ARDS) in 8%–15% of cases ((7,8,55,82–84)). Symptoms of acute pulmonary blastomycosis consist of fever, chills, headache, productive or non-productive cough, shortness of breath, chest pain and malaise ((12)). Symptoms of chronic pulmonary blastomycosis include fever, chills, persistent cough, hemoptysis, night sweats, decreased appetite and weight loss, which can be readily mistaken for the clinical signs of tuberculosis or cancer ((12)). Hematogenous dissemination occurs in 25%–40% of cases ((12)). Upon dissemination, any organ can potentially become involved, though the most commonly affected organ systems are the skin, followed by the bones and joints (e.g., long bones, thoracolumbar spine, ribs, skull), genitourinary tract (e.g., prostatitis, epididymo-orchitis) and central nervous system (CNS), where blastomycosis can present as meningitis, epidural abscesses, intracranial abscesses or other space-occupying lesions (i.e., granulomas) ((34,43,85–88)). Cutaneous disease may appear as single or multiple verrucous, nodular or ulcerative lesions on the face and distal extremities that are often marked by sharp, irregular borders, crusting and the formation of micro-abscesses in the underlying subcutaneous tissue ((43,89)).

The variety of clinical presentations of blastomycosis and their similarities to other conditions present challenges to early diagnosis. In a retrospective chart review, a median of 2.5 courses of antibiotics (interquartile range [IQR]: 1.5–4.5 courses) were prescribed prior to a diagnosis of pulmonary blastomycosis and a median of 23 days (IQR: 8–36 days) passed from the initial presentation to a healthcare facility before the correct diagnosis was made ((35)). Like pulmonary blastomycosis, cutaneous blastomycosis is often misdiagnosed as other pathologies such as basal cell or squamous cell carcinoma, keratoacanthoma, pyoderma gangrenosum (associated with autoimmune disease) or cutaneous tuberculosis ((7,76,90,91)). Osteomyelitis resulting from *Blastomyces* infection can mimic cancer (appearing as masses or lytic lesions on imaging) or skeletal tuberculosis ((7)). Further underpinning its reputation as a great masquerader, meningitis due to disseminated blastomycosis is frequently misdiagnosed as tuberculous meningitis, while blastomycosis-associated spinal or intracranial space-occupying lesions can be mistaken for malignancy ((7,86–88,92)).

A few studies have reported on the differences in clinical presentation and/or clinical severity between cases infected with *B. dermatitidis* compared to *B. gilchristii.* One study conducted in Québec, Canada found no association between *Blastomyces* genotype and the proportion of severe or fatal cases; however, only 2% of the patients in their sample were infected with *B. gilchristii*, reducing its power to discriminate clinically between species ((93)). A more recent study conducted in Wisconsin found that patients infected with *B. gilchristii* (n=80) were more likely to be hospitalized than those with *B. dermatitidis* (n=40), though the difference was no longer statistically significant following multivariate regression analysis (*p*=0.06) ((94)). Additional variation in clinical presentations between species was noted, where patients infected with *B. gilchristii* were more likely to present with fever (*p*<0.05) and those with *B. dermatitidis* had a significantly higher rate of developing disseminated infection with skin lesions (*p*<0.05) ((94)).

The mortality rate for blastomycosis was estimated in a 2020 systematic review and meta-analysis, which found an overall pooled mortality of 6.6% (95% CI: 4.9%–8.2%) across diagnosed cases of blastomycosis ((32)). This estimate is in relative agreement with studies from the US that estimated mortality to be 6.9%–10% ((36,49,68,71,95)); however, the US Centers for Disease Control and Prevention recently reported that the case fatality rate for blastomycosis across the five states where the disease was reportable rose to 17% in 2021, almost double the rate in 2019 ((96)). This sharp jump may be related to overwhelmed healthcare systems during the pandemic and patient hesitancy to seek medical care, which may have exacerbated diagnostic and treatment delays, leading to more severe disease presentations ((96)).

Risk factors associated with mortality and/or severe disease have included older age, immunosuppression, multi-lobar pulmonary involvement, ARDS and chronic disease (e.g., malignancy, chronic obstructive pulmonary disease, chronic lung disease, obesity, diabetes) ((32,49,68,93,97)). While the current evidence suggests that being immunocompromised is not a risk factor for developing blastomycosis, infection is more severe among those who are immunocompromised ((34)). The previously cited meta-analysis found that the pooled mortality rate was more than five times higher among patients who were immunocompromised (37%; 95% CI: 23%–51%) compared to general patients (6.6%; 95% CI: 4.9%–8.2%) ((32)). When complicated by ARDS, the mortality rate of blastomycosis reached 75% (95% CI: 53%–96%) ((32)).

### Laboratory diagnosis

The gold standard for the diagnosis of blastomycosis is by culture of sputum, tracheal aspirates, bronchoalveolar lavage fluid, cerebrospinal fluid, urine or biopsied tissue, but results can take 1–4 weeks ((34)). Microscopic visualization of yeast cells in smears or tissue specimens following the application of 10% potassium hydroxide and/or a fungal stain can offer a more rapid diagnosis, but this method is less sensitive than culture, so a negative result does not exclude a diagnosis of blastomycosis ((38)).

Serological tests have been available for decades, but their diagnostic value is limited by poor sensitivity, particularly early in infection and among patients who are immunocompromised ((38,81,98)). The most sensitive antibody test consisted of an enzyme immunoassay (EIA) that detected antibodies against *Blastomyces* adhesin-1 (BAD-1), a cell wall adhesion antigen and virulence factor. Despite a reported sensitivity of 88% and specificity of 94%–99%, this test has not gained widespread clinical use ((7,99)).

Enzyme immunoassays detecting a cell wall antigen known as galactomannan in patient body fluids have become a useful diagnostic tool that enables the rapid diagnosis of blastomycosis from a range of samples, including urine, serum, bronchoalveolar lavage fluid or cerebrospinal fluid ((8,12)). In the US, antigen EIA is a recommended component of diagnostic testing for suspected blastomycosis ((98,100)); however, in Canada, the adoption of antigen EIAs is reported to be limited ((81)). The sensitivity of antigen detection by EIA in urine samples of patients with proven disease is 76.3%–92.9% and the specificity is 79.3%, while the sensitivity of antigen EIA using serum as the sample source is somewhat lower, ranging from 56%–82% ((98,100–103)). False-positives can occur due to cross-reactivity with other fungal pathogens, particularly *Histoplasma*, which can be an issue in areas where their respective geographic distributions overlap; fortunately, the recommended treatments for histoplasmosis and blastomycosis are similar, reducing the harm of misdiagnosis ((5,100,101,104,105)). Note that serologic testing may be helpful for differentiating blastomycosis from histoplasmosis and on occasions when antigen EIAs are negative but suspicion for blastomycosis remains ((98)). It has been suggested that antigen EIA levels may correlate with disease severity and could be used to monitor the response to blastomycosis treatment, but at least with respect to antigen testing for histoplasmosis, low levels of antigenuria may persist in some patients for months even after successful eradication ((8,34,104,106,107)).

Advances in the molecular diagnosis of *Blastomyces* infection could help facilitate the workup of patients with possible blastomycosis and reduce diagnostic delays; however PCR-based tests are currently limited to reference laboratories and have not been widely standardized ((7,34,106)).

Given the imperfect sensitivity and specificity of currently available tests, no single test is sufficiently accurate for diagnosis in isolation ((100)). In combination with clinical and epidemiological history, physicians often need to use multiple diagnostic methods to get an acceptable level of diagnostic accuracy ((98,100)).

### Treatment

All patients diagnosed with blastomycosis should receive antifungal therapy regardless of the clinical presentation because of the risk of progression or recurrence of symptoms if left untreated ((34)). Mild to moderate blastomycosis is treated with oral itraconazole, while moderate to severe blastomycosis is initially treated with lipid formulations of amphotericin B for 1–2 weeks until clinical improvement is noted (4–6 weeks in the case of CNS disease), following which, therapy is completed using oral itraconazole ((34,104)). Total treatment duration is typically 6–12 months and depends on the severity of the infection, immune status of the host and involvement of the bone, joints or CNS ((7,34,104)). Lifelong suppressive therapy with oral itraconazole may be required for immunosuppressed patients if immunosuppression cannot be reversed ((104)).

Several other azoles have been used to treat blastomycosis; however, clinical trial data for these other agents is still limited. For example, a novel formulation of itraconazole is available, called super-bioavailability itraconazole (SUBA-itra), which exhibits enhanced intestinal absorption relative to the conventional formulation with reduced inter-patient pharmacokinetic variability and a lower impact from food and alterations in gastric acidity ((108)). Voriconazole, a relatively newer azole, has been shown to be effective as an alternative to itraconazole when there is CNS involvement due to its better penetration of the blood brain barrier and excellent *in vitro* activity against *B. dermatitidis*, followed by fluconazole, which also demonstrates good penetration into the CNS and is moderately effective against blastomycosis ((8,92,104)). Another relatively newer azole, posaconazole, has been used to treat non-CNS blastomycosis, as it penetrates the blood brain barrier poorly but may have more potent fungicidal activity against *Blastomyces* than itraconazole as well as improved oral absorption and fewer adverse effects ((8,109)). Limited data for oral isavuconazole also exist ((7,8,110)). Given their narrow therapeutic windows, therapeutic drug monitoring is recommended when using either itraconazole, voriconazole or posaconazole in order to ensure adequate steady-state serum levels and avoid drug-related toxicities ((111)).

Pregnant patients who are diagnosed with blastomycosis should receive intravenous liposomal amphotericin B without a subsequent azole, due to the teratogenic effects of azoles, until after delivery or until resolution of the infection, whichever occurs first ((7)). Post-delivery, the placenta should be examined for signs of *Blastomyces* infection, the newborn monitored closely and amphotericin B deoxycholate given if the newborn is found to be infected ((7)).

### Impacts of climate change

The future climate projected for Canada includes increasing temperatures, increasing rainfall (although with more regional variation than for temperature) and a greater fraction of precipitation during the winter occurring as rain rather than snow ((13,112)). The climate is also expected to become more variable, with an increase in extreme weather events, including heat waves, summertime droughts and more frequent and severe storms that are expected to increase the possibility of flooding ((13,112)). Risks from fungal infections such as blastomycosis are likely to change with these expected changes in temperature and rainfall ((4,13)). Globally, over half of known infectious diseases (n=218/375) were found to be potentially exacerbated by the effects of climate change, including blastomycosis and twenty-three other fungal diseases ((113)). Climate change may also drive changes to the geographic ranges of several dimorphic fungi that are endemic to North America, such as *Coccidioides* in Southwestern US and *Histoplasma* and *Blastomyces* in the US and Canada ((3,4,114–116)). These changes may be long-lasting or, perhaps, transient and variable. A study conducted in Minnesota reported the transient detection of *Blastomyces* DNA in environmental samples obtained from a non-endemic location following a flood, pointing, once again, to the sometimes evanescent nature of this fungal pathogen when short-term climactic conditions transiently favour fungal growth in otherwise non-conducive environments ((117)). Climate change may also impact the frequency of cases, and an association between flooding and frequency of blastomycosis cases has been described in the literature ((117–120)).

Greer *et al.* speculated that the dry summers and heavy wintertime precipitation projected for North America would provide optimal conditions for the dispersal of *Blastomyces* spores ((121)). Panackal further pointed out that a common pattern emerges among fungal species, in which growth is facilitated by soil moisture from precipitation and humidity, followed by a dry period when fungal hyphae desiccate and form spores, culminating with windy conditions, potentially in the form of storms, hurricanes and tornados, which then aerosolize and disperse the spores over great distances ((122)). Among several blastomycosis case clusters reported in the literature, exposure was considered to have occurred following periods of diminished precipitation or drought and in association with rainfall events, reinforcing the significance of dry and wet cycles ((38,55–57,61–63,123)). During abnormally dry days, whose frequency, intensity and duration will increase as the climate continues to grow warmer, winds can disperse fungal spores in the same way that they spread pollen ((13)).

## Conclusion

This review summarizes the evidence regarding blastomycosis in Canada and highlights what is currently known and where important knowledge gaps exist for this fungal disease. While projected to have a larger public health impact due to climate change-associated shifts in temperature and rainfall, our understanding of the interplay between blastomycosis and environmental factors is far from complete ((2–4,13,121–128)). Further study of *Blastomyces’* ecological niche and climate sensitivity will require novel approaches and greater laboratory test capacity to isolate the pathogen from the environment ((129)). This could, in turn, facilitate improved modelling of the potential impacts of climate change on risks to the public and help inform more effective measures for prevention ((38)).

Bearing in mind the uncertainties concerning the current and future epidemiology of blastomycosis, the most important factor in limiting morbidity and mortality is increasing healthcare providers’ awareness of blastomycosis to ensure timely diagnosis and treatment and prevent more serious progression of disease. In the event of outbreaks, early clinical recognition also leads to the early engagement of public health authorities, who can introduce measures to limit ongoing exposures and prevent new cases. Finally, increased awareness among the public living in endemic/hyperendemic areas, particularly those at higher risk, such as Indigenous Peoples and individuals who are immunocompromised, can empower them to ask their healthcare providers about the possibility of blastomycosis should they develop compatible symptoms.

In addition to promoting greater education and awareness, other key measures to consider include the following: funding research to close knowledge gaps regarding the ecology of *Blastomyces*; improving surveillance by making blastomycosis more widely reportable; developing rapid, accurate and standardized PCR-based assays to improve timely case detection; exploring the potential of computer-aided diagnosis using artificial intelligence and machine learning models that could prompt physicians to consider blastomycosis early in the diagnostic process ((111,130,131)); and finally, developing safer and better-tolerated antifungal treatments to maximize treatment adherence, especially in view of the prolonged length of therapy that can be required for this disease.
